# Physical fitness in adults with isolated secundum atrial septal defects^☆^

**DOI:** 10.1016/j.ijcchd.2025.100632

**Published:** 2025-10-24

**Authors:** Linda Ashman Kröönström, Anna-Klara Zetterström, Mikael Dellborg, Åsa Cider, Peter Eriksson, Emelie Dahlin, Zacharias Mandalenakis

**Affiliations:** a Occupational and Physical Therapy Department, Sahlgrenska University Hospital, Gothenburg, Sweden; b Institute of Neuroscience and Physiology/Physiotherapy, Sahlgrenska Academy, University of Gothenburg, Gothenburg, Sweden; c Adult Congenital Heart Disease Unit, Sahlgrenska University Hospital, Östra Hospital, Gothenburg, Sweden; d Institute of Medicine, Sahlgrenska Academy, University of Gothenburg, Gothenburg, Sweden

**Keywords:** Atrial septal defect, Adult congenital heart disease, Congenital heart disease, Muscular fitness, Muscle strength, Rehabilitation

## Abstract

**Background:**

Atrial septal defect (ASD) is one of the most common diagnoses in patients with adult congenital heart disease (ACHD). However, recent studies indicate that ASD is not so benign and may impede physical fitness. We aimed to assess physical fitness in patients with isolated secundum ASD compared with healthy reference values and to evaluate the results according to the absence or presence of repair.

**Methods:**

Between April 2009 and March 2025, patients with ACHD performed physical fitness tests comprising five muscular fitness tests and one cardiorespiratory endurance test. Data were stored in a registry at the Physical Therapy Department, Sahlgrenska University Hospital/Östra. In total, 102 adults with isolated secundum ASD were included in this descriptive, register-based cohort study.

**Results:**

Both women and men with ASD showed significantly lower isoinertial muscular fitness test results (heel lifts: p < 0.001 and shoulder flexions: p < 0.001) and maximal exercise capacity (p < 0.001) compared with healthy reference values. The shoulder flexion test revealed the lowest percentage of reference values for women and men with ASD (58.3 % and 53.3 %, respectively). Women who had undergone surgical procedures performed the timed-stands test significantly faster when compared with women who had undergone device procedures (13.0 s [10.2, 16.0] vs. 19.1 s [15.1, 26.2]; p = 0.014).

**Conclusion:**

Patients with less complex ACHD, such as secundum ASD, may have impaired muscular fitness and cardiorespiratory endurance. Therefore, all patients with ACHD could be offered tests of physical fitness. These tests are necessary when prescribing individualized exercise, as suggested by guidelines.

## Highlights

• Patients with secundum ASD may have impaired muscular fitness.
• Patients with secundum ASD may have impaired cardiorespiratory endurance.
• All patients with ACHD could be offered tests of physical fitness.
• Tests of physical fitness are necessary when prescribing individualized exercise.

## 1 Introduction

The prevalence of adult congenital heart disease (ACHD) is increasing due to improvements in surgery and care [1,2], as well as in the detection of mild lesions [3]. Congenital heart disease (CHD) consists of almost 50 different diagnoses in a wide variety of combinations, resulting in a wide range in complexity. Atrial septal defect (ASD) is one of the most common CHD diagnoses [4], and it can exist as an isolated heart defect or in combination with other CHD diagnoses as a complex form of the CHD. It is not uncommon for patients with ASD to be asymptomatic and undiagnosed until adulthood [5]. The survival rate in patients with ASD is lower than in the general population; however, a catheter or surgical closure during childhood has been found to improve survival [6]. The care of patients with isolated ASD sometimes terminates after the closure of the defect, and patients may be clinically considered cured. The complexity of the defect varies between different types of ASD (primum and secundum defects, as well as inferior and superior sinus venosus defects), and secundum ASDs are considered the most benign types [7]. Although an ASD is often considered less complex or a mild form of CHD [7,8], recent studies indicate that patients with these defects have coexisting ailments, such as increased risk of atrial fibrillations, strokes [9], premature mortalities and chronic diseases [10]. Depression and anxiety have also been reported [11].

As patients live longer with CHD, parameters studying lifestyle factors are becoming increasingly important. Physical fitness and level of physical activity are important components of these parameters. Patients with ASD have decreased maximal oxygen consumption (VO_2max_) [12,13]. This is observed even in allegedly asymptomatic patients [14]. A previous study showed that patients with a small unrepaired or spontaneously corrected ASD have impaired VO_2peak_ compared with healthy reference values [15]. Muscular fitness tests in patients with ACHD have shown decreased isometric [16,17] and isoinertial values [18]. In male conscripts, later diagnosed with ASD and intervened, muscular fitness assessment results based on handgrip strength, elbow flexion and knee extension were not significantly decreased compared with a reference population [19]. However, no previous studies have specifically reported muscular fitness in patients with isolated secundum ASD. We hypothesised that the absence or presence of repairs may impact physical fitness. Thus, patients who have undergone corrective cardiac surgery may have more severe ASD and thus more impaired physical fitness than patients who have noncorrected ASD. Therefore, we aimed to compare the physical fitness test results of patients with isolated secundum ASD with healthy reference values. In addition, we assessed the physical fitness test results according to the absence or presence of repair.

## 2 Methods

### 2.1 Study population

This was a descriptive, register-based cohort study. Generally, patients at the ACHD unit, Sahlgrenska University Hospital/Östra are offered tests of physical fitness as an add-on to ordinary care. These tests have been offered since 2009 and are, as far as possible, offered in connection with patients’ visits to the ACHD unit. Patients are excluded from tests of physical fitness for any of the following reasons: intellectual disabilities making it difficult to perform tests, severe psychiatric disorders, unstable or severe cardiac illness or discontinuation of planned care. Patients were orally informed and asked if they agreed to keep their physical fitness test results in a registry. Patients who agree to this provide a written informed consent document.

Patients with a primary International Classification of Diseases (ICD)-10 diagnosis code for ASD (Q211) were retrieved from the registry. Thereafter, all patients with an ICD-10 code Q211 were manually reviewed, and those with any of the following diagnoses were excluded from the analyses: a patent foramen ovale (PFO), primum ASD and sinus venosus ASD (which all have the same ICD-10 code). Patients were included in the present registry study if their primary diagnosis was an isolated ICD-10 code secundum ASD (Q211). If patients had performed multiple physical fitness tests between April 2009 and March 2025, the test performed on the first visit was chosen for the assessment. Data regarding medication, the presence of repair and New York Heart Association (NYHA) functional classification [20], were retrieved from the patient's medical record. The study complied with the ethical guidelines of the Declaration of Helsinki and was approved by the Swedish ethical review authority (DNR 2021–02149).

**Fig. 1:**
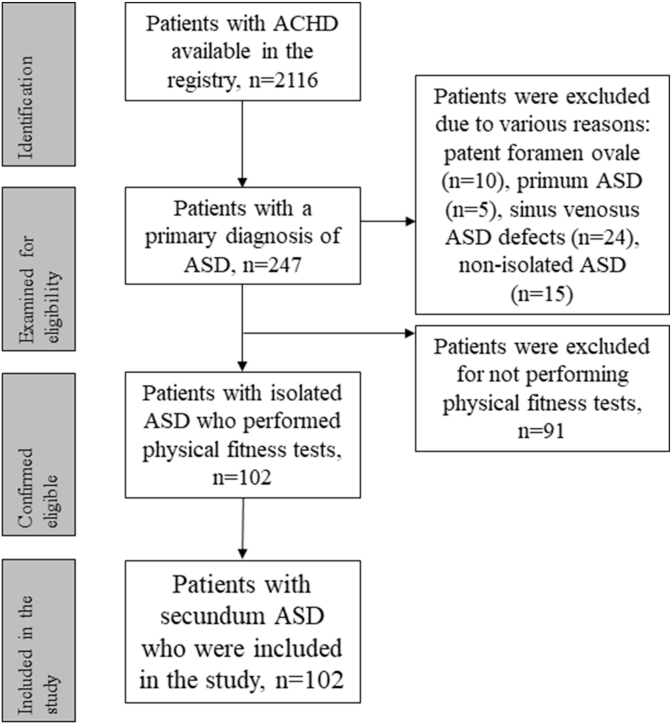
**Flowchart of the distribution of patients during the recruitment** ACHD: adult congenital heart disease, ASD: atrial septal defect.

Of the 247 patients in the registry with a primary diagnosis of ICD-10 code Q211, 10 patients (4.0 %) had PFOs, 5 patients (2.0 %) had primum ASDs, 24 patients (9.7 %) had sinus venosus ASDs, and 15 patients (6.1 %) had a non-isolated ASD. Therefore, these patients were removed from the analyses. Furthermore, 91 patients (36.8 %) did not perform physical fitness tests. This resulted in 102 patients (41.3 %) with ASD who matched the inclusion criteria. The patient inclusion flowchart is depicted in Fig. 1.

### 2.2 Measurements

Muscular fitness was assessed in the upper and lower extremities using the following three isoinertial (iso [same] and inertial [resistance] throughout the concentric and eccentric phases of muscle contraction [21]) tests: heel lift, shoulder flexion and timed-stands test (TST). Furthermore, two isometric (a muscle action without noticeable change in muscle length [22]) tests – handgrip strength and shoulder abduction – were used to assess muscular fitness. To evaluate cardiorespiratory endurance, a symptom-limited submaximal ergometer bicycle test was used [23]. The execution of these tests in patients with ACHD has previously been published [18,24,25].

#### 2.2.1 Reference values

The results of the muscular fitness and bicycle tests among the participants were compared with the available published reference values described for each respective test. Where there were no reference values for an entire age span, the patients were grouped together with the nearest age group.

#### 2.2.2 Isoinertial tests

Heel lifts were performed on a 10° wedge using the right and left legs, each with the contralateral foot held slightly above the floor. Participants were allowed to touch the wall for balance and were instructed on each rise to touch their heads to a marker on a measuring stick that was calibrated in advance. The speed was set to 30 contractions/min using a metronome (Wittner Taktell Piccolo, Germany), and the maximum number was registered. The reference group consisted of healthy volunteers living in Sweden: n = 566, aged 20–81 years [26].

Shoulder flexion was performed with the participant sitting on a stool, back touching the wall and with a hand-held weight in the hand of the arm to be tested (3 kg for men, and 2 kg for women). The participant was asked to elevate the testing arm from 0 to 90° flexion a maximal number of times at a speed of 20 contractions/min using a metronome (Wittner Taktell Piccolo, Germany). The reference group consisted of 20 healthy persons, aged 70.5 ± 5.9 years living in Sweden and 13/20 (65 %) were men. These values were not presented according to sex [27].

The TST measures muscular fitness in the lower extremity [28]. The participant sat on a 45- cm-high stool without armrests, wearing shoes and with arms placed on the chest. Then, the participant was urged to perform 10 stand-and-sit actions as fast as possible. The completion time in seconds was noted. The reference group consisted of healthy subjects living in the US: n = 139, aged 20–85 years [28].

#### 2.2.3 Isometric tests

Handgrip strength was measured using a Jamar dynamometer (Sammons Preston Rolyan, Chicago, USA), which measures the maximum handgrip in the cylindrical grip. The participant sat with adducted shoulders on a chair without armrests. The elbow of the tested hand was held at 90°. The mean value of the three repeated tests was used for the grip strength calculations. The reference group consisted of healthy volunteers living in the US: n = 628, aged 20–75+ years [29].

Shoulder abduction was assessed using IsoForce Control®, a portable dynamometer (Medical Device Solutions, Oberburg, Switzerland). The participants sat on a stool, back touching the wall and legs stretched forward and crossed, with one heel touching the floor. The participant's arm was elevated to 90° in the scapula direction, and a strap was placed around the wrist (styloid process) on the dominant arm. The mean of the three repeated tests results was used to calculate the shoulder abduction value. The reference group consisted of healthy individuals living in Sweden: n = 60, aged 19–57 years [30].

#### 2.2.4 Cardiorespiratory endurance

A symptom-limited submaximal ergometer bicycle protocol [23] was used with an initial load of 25 or 50 W and stepwise increases every 4.5–5 min. Heart rate, blood pressure, ratings of perceived exertion (RPE) [31] and oxygen saturation were registered before starting and continuously throughout the test. The test ended when patients reached an RPE of 15–17 (given that all other measurements were considered normal). Because not all patients completed an entire watt load of 4.5 or 5 min, calculations according to Strandell were performed to obtain a specific watt level for each patient [32]. To enable comparisons between submaximal values and maximal reference values, corrections were performed in several steps, as described in a previous study [25]. The reference group consisted of individuals performing tests at the department of Clinical Physiology in Kalmar, Sweden with results considered as normal by the attending physician: n = 1790, aged 7–80 years [33].

#### 2.2.5 Level of physical activity

Patients reported their self-assessed level of physical activity during the preceding week using the International Physical Activity Questionnaire (IPAQ) – short form [34].

### 2.3 Statistical methods

Data were analysed using the Statistical Package for Social Sciences (SPSS) 29.0 (IBM Corp., Armonk, NY, USA). Demographic data are presented as mean values (standard deviation [SD]) or median values (interquartile range [IQR]). Patients were divided into the following three groups: *corrective cardiac surgery* (included patients who had undergone device surgery as well as corrective surgery), *device closure* and *nonoperated patients*. The Shapiro–Wilk test, Kolmogorov–Smirnov test and Q-Q plots were used to test for normality. The tests of normality resulted in comparisons between groups being assessed using a parametric student's t-test or, if non-normally distributed, a nonparametric Mann–Whitney *U* test. When analysing more than two independent groups, a parametric one-way analysis of variance (ANOVA) was used. For nonparametric calculations, the Kruskal–Wallis test was used. The Chi-square test was used for analysis of categorical data. The one-sample *t*-test was used to analyse the values of patients with ASD in comparison with healthy reference values, and the one-sample Wilcoxon signed rank test was used for nonparametric data. Tests were two-sided, and *p* < 0.05 was considered to represent statistical significance.

Body mass index (BMI) was calculated as weight in kg divided by height in m squared. BMI is classified by the World Health Organization (WHO) as follows: underweight <18.5, normal weight 18.5–24.9, pre-obesity 25–29, obesity class I 30–34.9, obesity class II 35–39.9 and obesity class III ≥40 [35]. Physical fitness results are presented as percentages of reference values, mean values (SD) and median values [IQR]. The IPAQ was used as a descriptive variable of patients’ levels of physical activity, and data were analysed and presented as median values [IQR] according to guidelines [34].

## 3 Results

### 3.1 Demographics

The majority of the participants were women (n = 73, 71.6 %). The median age of the participants was 43.5 [29.7, 61.0] years (Table 1).

**Table 1:** Characteristics of the study population.

	All patients n = 102	Women n = 73	Men n = 29
Age, years, median [IQR]	43.5 [29.7, 61.0]	45.0 [30.0, 63.0]	43.0 [25.0, 56.0]
Height, cm	170.2 (9.6)	166.3 (7.9)	180.0 (5.9)
Weight, kg, median [IQR]	72.0 [64.7, 84.2]	69.0 [60.5, 78.5]	84.0 [76.0, 99.0]
BMI, kg/m^2^, median [IQR]	25.2 [22.5, 28.6]	24.4 [22.1, 28.1]	25.7 [24.6, 30.3]
BMI classification			
under weight (<18.5), yes	1 (1.0)	1 (1.4)	0 (0.0)
normal weight (18.5–24.9), yes	47 (46.1)	38 (52.1)	9 (31.0)
pre-obesity (25–29.9), yes	35 (34.3)	23 (31.5)	12 (41.4)
obesity class I (30–34.9), yes	16 (15.7)	9 (12.3)	7 (24.1)
obesity class II (35–39.9), yes	2 (2.0)	2 (2.7)	0 (0.0)
obesity class III (≥40), yes	1 (1.0)	0 (0.0)	1 (3.4)
Cardiovascular medication, yes	53 (52.0)	39 (53.4)	14 (48.3)
NYHA classification I/II/III–IV	88/14/0	59/14/0	29/0/0
Systolic blood pressure, mmHg	121.9 (17.8)	119.7 (17.6)	127.4 (17.4)
Diastolic blood pressure, mmHg	71.1 (9.7)	69.7 (9.0)	74.3 (10.6)
IPAQ classification	n = 74	n = 56	n = 29
Low/Moderate/High, n	20/36/18	15/31/10	5/5/8
MET, min/week, median [IQR]	1404 [693.0, 2958.0]	1386.0 [693.0, 2664.0]	2153.0 [960.0, 6014.2]

Data are given as n (%) or mean (standard deviation [SD]) unless otherwise noted. ASD: atrial septal defects, BMI: body mass index, NYHA: New York Heart Association classification, IPAQ: International Physical Activity Questionnaire, MET: Metabolic equivalent of task, IQR: interquartile range.

### 3.2 Physical fitness in adults with secundum ASD compared with healthy reference values

Both women and men with ASD showed significantly lower isoinertial muscular fitness test results (heel lifts: p < 0.001 and shoulder flexions: p < 0.001) and maximal exercise capacity (p < 0.001) compared with healthy reference values, respectively (Table 2). Of all tests of muscular fitness, the shoulder flexion test in both women and men with ASD constituted the lowest percentage (women: 58.3 [IQR 40.0, 85.0] %; men: 53.3 [IQR 37.5, 70.8] %) compared with reference values.

**Table 2:** Physical fitness test results in men and women with secundum ASD compared with healthy reference values.

*Measurement property*	Women				Men			
Test	with ASD	Reference values∗	%	p-value	with ASD	Reference values∗	%	p-value
***Cardiorespiratory endurance***								
**Maximal exercise capacity, watt**	n = 58				n = 25			
Mean	109.9 (31.7)	152.7 (23.4)	72.9 (21.8)	**<0.001**^a^	163.9 (48.9)	252.3 (24.7)	65.6 (21.4)	**<0.001**^a^
Median	109.5 [88.8, 130.8]	161.5 [131.5, 175.0]			162.0 [132.5, 200.5]	260.3 [229.8, 270.4]		
***Muscular fitness – Isoinertial***								
**Heel lift, right, n**	n = 61				n = 29			
Mean	17.6 (8.0)	25.4 (4.8)	70.3 (33.6)	**<0.001**^a^	22.2 (9.1)	29.6 (6.7)	79.9 (45.8)	**<0.001**^a^
Median	18.0 [11.5, 24.0]	28.0 [19.9, 30.7]			22.0 [15.5, 30.0]	28.5 [24.0, 37.5]		
**Shoulder flexion, n**	n = 73				n = 29			
Mean	36.7 (20.0)	c			43.3 (18.6)	c		
Median	32.0 [24.0, 45.0]	60.0 [c]	58.3 (40.0, 85.0)	**<0.001**^b^	40.0 [25.5, 61.0]	60.0 [c]	53.3 (37.5, 70.8)	**<0.001**^b^
**TST, s**	n = 43				n = 12			
Mean	17.6 (8.6)	15.0 (2.8)	98.0 (33.5)		15.4 (2.6)	13.0 (3.5)	86.1 (25.4)	
Median	15.2 [10.8, 24.0]	14.3 [12.6, 17.6]		0.085^b^	15.0 [13.2, 17.0]	12.2 [10.0, 15.6]		0.065^b^
***Muscular fitness – Isometric***								
**Handgrip strength, lbs.**	n = 68				n = 28			
Mean	61.2 (16.8)	65.5 (8.9)	94.9 (33.0)	0.061^b^	103.2 (18.0)	111.6 (13.9)	94.2 (20.5)	0.096^b^
Median	62.4 [49.5, 71.7]	68.1 [55.1, 74.1]			104.3 [90.9, 117.4]	116.8 [103.3, 121.0]		
**Shoulder abduction, kg**	n = 61				n = 28			
Mean	5.0 (1.2)	4.7 (0.0)	105.5 (24.7)	0.091^b^	8.4 (2.2)	9.2 (0.0)	91.9 (24.4)	0.065^b^
Median	5.0 [4.2, 5.9]	4.7 [4.7, 4.7]			8.6 [7.3, 9.9]	9.2 [9.2, 9.2]		

Mean values are presented as standard deviations (SDs) and median values as interquartile ranges (IQRs). ASD: Arterial septal defect, TST: timed-stands test.

Higher values indicate greater physical fitness, with the exception of the TST in which the opposite is valid.

a One Sample *t*-test.

b One Sample Wilcoxon Signed Rank Test.

c Not available.

### 3.3 Physical fitness in adults with secundum ASD according to the absence or presence of repair

There was a significant difference in the proportion of patients using cardiovascular medications between the groups according to the absence or presence of repair (Supplementary Table 1). Women who had undergone surgical procedures performed the TST test significantly faster when compared with women who had undergone device procedures (13.0 s [10.2, 16.0] vs. 19.1 s [15.1, 26.2]; p = 0.014) (Table 3). There were no significant differences in physical fitness in men between the groups according to the absence or presence of repair.

**Table 3:** Physical fitness test results in men and women with secundum ASD according to the absence or presence of repair.

Sex	Test	Patients who have undergone corrective surgery	Patients who have undergone device closure	Non-operated patients	p-value surgery vs. device	p-value device vs. non-operated	p-value surgery vs. non-operated
**Women**	**Heel lift, n**	n = 23	n = 19	n = 19			
	Mean	17.4 (8.5)	15.9 (8.8)	19.5 (6.6)	0.595^a^	0.282^a^	0.074^a^
	Median	15.0 [11.0, 24.0]	17.0 [11.0, 24.0]	19.0 [17.0, 24.0]			
	**Shoulder flexion, n**	n = 28	n = 22	n = 23			
	Mean	36.4 (23.7)	31.5 (12.6)	42.1 (20.3)			
	Median	30.5 [20.7, 47.0]	35.0 [21.7, 41.7]	32.0 [28.0, 55.0]	0.941^b^	0.325^b^	0.399^b^
	**Timed-stands test, s**	n = 15	n = 10	n = 18			
	Mean	13.7 (4.2)	22.1 (8.7)	18.3 (10.2)			
	Median	13.0 [10.2, 16.0]	19.1 [15.1, 26.2]	14.6 [10.6, 24.7]	**0.014**^b^	0.318^b^	0.483^b^
	**Handgrip strength, lbs.**	n = 24	n = 21	n = 23			
	Mean	59.9 (15.4)	60.7 (19.4)	62.9 (16.3)			
	Median	61.1 [48.2, 70.2]	61.7 [52.9, 69.4]	66.1 [44.1, 73.4]	0.757^b^	0.717^b^	0.511^b^
	**Shoulder abduction, kg**	n = 25	n = 18	n = 18			
	Mean	5.2 (1.0)	5.0 (1.5)	4.6 (1.0)			
	Median	5.1 [4.4, 5.9]	5.0 [4.2, 6.2]	4.8 [3.7, 5.4]	0.754^b^	0.224^b^	0.144^b^
	**Submaximal exercise capacity, watt**	n = 24	n = 17	n = 17			
	Mean	89.8 (24.3)	76.5 (27.0)	89.1 (34.3)	0.858^a^	0.503^a^	0.350^a^
	Median	93.6 [72.2, 107.6]	75.0 [51.4, 98.6]	86.1 [72.2, 105.6]			
**Men**	**Heel lift, n**	n = 10	n = 10	n = 9			
	Mean	18.3 (7.0)	24.1 (11.0)	24.3 (8.5)	0.083^a^	0.218^a^	0.491^a^
	Median	19.5 [13.0, 23.2]	26.5 [15.7, 32.0]	26.0 [15.5, 29.5]			
	**Shoulder flexion, n**	n = 10	n = 10	n = 9			
	Mean	38.0 (19.2)	46.8 (18.7)	45.3 (18.7)			
	Median	35.5 [19.0, 60.0]	49.5 [31.5, 64.0]	43.0 [29.0, 55.0]	0.297^b^	0.607^b^	0.224^b^
	**Timed-stands test, s**	n = 5	n = 3	n = 4			
	Mean	16.3 (3.2)	15.3 (2.9)	14.3 (1.8)			
	Median	15.0 [14.0, 19.3]	17.0 [12.0, c]	14.5 [12.5, 15.9]	0.533^b^	0.333^b^	0.267^b^
	**Handgrip strength, lbs.**	n = 10	n = 10	n = 8			
	Mean	97.9 (21.8)	113.5 (12.7)	97.1 (13.9)			
	Median	98.9 [86.6, 113.5]	116.4 [103.4, 122.9]	98.1 [89.8, 104.9]	0.077^b^	0.145^b^	0.456^b^
	**Shoulder abduction, kg**	n = 10	n = 10	n = 8			
	Mean	7.7 (2.3)	9.1 (1.6)	8.5 (2.9)			
	Median	7.7 [5.7, 10.0]	8.9 [7.9, 10.1]	8.4 [6.0, 9.9]	0.161^b^	0.955^b^	0.388^b^
	**Submaximal exercise capacity, watt**	n = 9	n = 9	n = 7			
	Mean	102.8 (42.6)	145.4 (44.8)	125.1 (21.7)	0.630^a^	0.092^a^	0.299^a^
	Median	100.0 [72.2, 140.3]	147.2 [106.9, 188.9]	122.2 [103.1, 150.0]			

IQR: interquartile range, No: number of patients, NA: not available.

Higher values indicate greater physical fitness, with the exception of the TST in which the opposite is valid.

a Student's t-test.

b Mann–Whitney *U* test.

c Not available.

## 4 Discussion

The results of the present study show that both women and men with isolated secundum ASD had lower isoinertial muscular fitness in two of the three tests (heel lifts: p < 0.001 and shoulder flexions: p < 0.001) when compared with healthy reference values. Furthermore, the shoulder flexion test revealed the lowest percentage of reference values for women and men with ASD (58.3 % and 53.3 %, respectively). Male military conscripts who later had interventions because of ASD were found not to have decreased muscular fitness compared with a reference population [19]. However, no previous studies have assessed muscular fitness in women with ASD, despite investigations of different entities of muscular fitness in the broad group of patients with ACHD. In a previous study, decreased isoinertial muscular fitness was associated with increased CHD severity in patients with ACHD compared with healthy controls [18]. The shoulder flexion test results for women and men in this study were the lowest of the reference values. Regarding isometric muscular fitness, a previous study showed decreased muscular fitness in adults with complex ACHD [16].

In the present study, men and women with secundum ASD showed decreased cardiorespiratory endurance compared with healthy reference values, and these results corroborate previous studies that also showed decreased VO_2max_ [12,13,15]. The mechanisms of decreased VO_2max_ are reduced left ventricular filling and stroke volume [36], as well as an abnormal increase in pulmonary artery pressure during exercise [37]. Depending on the severity and size of the defect, an ASD can remain undiagnosed until adulthood. This may be due to ageing, stiffening of the left ventricular myocardium, which causes an increase in the left-to-right shunt with more haemodynamic significance [7]. We hypothesised that patients with ASD who were diagnosed at birth or during childhood may have had restrictions on physical activity and exercise. These patients may have been discouraged from exercising, as opposed to patients who have lived without knowledge of their CHD. We also hypothesised that the absence or presence of repairs may impact physical fitness. The results of the present study indicated that women who had undergone surgical procedures performed the TST test significantly faster when compared with women who had undergone device procedures; however, no other significant results were found according to the absence or presence of repair. In the present study, 72.9 % of the patients had undergone reconstructive surgery. Today, device closure is the first treatment of choice for patients with secundum ASD [5].

Other components of reduced physical fitness may be explained in part by a lack of physical activity. Nowadays, patients with ACHD are encouraged to be physically active and to perform exercise at individualized levels [38,39]; however, this has not always been the case. In the present study, women reported a MET value of 1386.0 [693.0, 2664.0] min/week, while men reported a MET value of 2153.0 [960.0, 6014.2]. To assess different aspects of physical fitness in patients with ACHD, particularly as patients currently live with their condition for longer periods of time, may be considered an integral part of care of patients with ACHD. BMI may be related to low physical fitness and low levels of physical activity and may play a role as a risk factor for developing cardiovascular disease and mortality. Thus, it may be important to assess it in the present population. The disadvantage of using BMI is its inability to discriminate between muscle and fat masses. Since a fit person has more muscle than fat mass, a high BMI may not always be a sign of obesity. A meta-analysis assessed the associations between BMI, cardiorespiratory fitness and mortality and showed that cardiorespiratory fitness is a more important risk indicator than fatness [40].

Patients with ASD are mostly females, with the proportion ranging from 60 % [4] to 65–75 % [5]. In the present study, the proportion of female patients was 71.6 %, which is within the range noted in past studies. Secundum ASD accounts for 80 % of these defects [7]. Because of the heterogeneity between different forms of ASD, we focused only on secundum ASDs.

To be able to prescribe individualized exercise, tests of physical fitness are essential [41]. The results of the present study have implications for clinical practice in terms of highlighting the usefulness of tests of peripheral function (i.e., muscular fitness) in all patients with ACHD, including patients with lesions considered to be less complex, such as secundum ASD. Peripheral factors are a key component of physical fitness and may therefore be included in regular evaluations of physical fitness, as well as in actual exercise prescriptions. The lifelong follow-up of patients with ASD regardless of age at diagnosis, size of defect and whether ASD is closed, which has been suggested by others [10], may therefore include the assessment of peripheral factors.

### 4.1 Strengths and limitations

The strengths of the present study include the number of patients with isolated secundum ASD, its clinical relevance and the novel area of muscular fitness in this population, which has not previously been assessed. The study also has the following limitations. Patients with the simplest forms of ASD may not always be followed at an ACHD centre; therefore, there may be a risk of selection bias in the studied population resulting in the most sick patients being followed at the ACHD-centre. The lack of motivation could have contributed to patients opting to decline to take part in tests of physical fitness. Not all patients performed all tests, and the reasons for this were not noted. However, in the clinical setting, this is common and can be the result of many factors, such as musculoskeletal disabilities in specific parts of the body or lack of time, and it is likely for one to believe that these tests were missed at random and not systematically. The TST was not in use at our centre until 2013; therefore, fewer patients performed this test compared with other tests. The reference values for the shoulder flexion test did not represent the entire age span of the included population, and the reference values were not presented according to sex, which is a limitation. This may result in women with ASD presenting lower values than the reference values, which consists of both men and women, and the contrary for men. A group of matched controls performing all tests would have been preferred, but this was not available in the clinical setting. Furthermore, none of the tests of muscular fitness have been assessed regarding validity and reliability for this population. Patients with PFO were excluded due to the different nature of this condition (no missing septal tissue). The group of patients with PFO usually resembles a larger proportion of the population of patients with ASD; however, these patients are often omitted once the PFO is closed, which may explain why these patients were not referred to the physiotherapy clinic and thus were not included in the present registry. Finally, when divided into groups according to the absence or presence of repair, the number of participants in each group was limited.

## 5 Conclusion

Patients with less complex ACHD, such as secundum ASD, may have impaired muscular fitness and cardiorespiratory endurance. Therefore, all patients with ACHD could be offered tests of physical fitness. Tests of physical fitness are necessary when prescribing individualized exercise, as suggested by guidelines.

## CRediT authorship contribution statement

**Linda Ashman Kröönström:** Writing – review & editing, Writing – original draft, Methodology, Funding acquisition, Formal analysis, Data curation, Conceptualization. **Anna-Klara Zetterström:** Writing – review & editing, Methodology, Funding acquisition, Formal analysis, Data curation, Conceptualization. **Mikael Dellborg:** Writing – review & editing, Methodology, Funding acquisition, Formal analysis. **Åsa Cider:** Writing – review & editing, Methodology, Formal analysis, Data curation. **Peter Eriksson:** Writing – review & editing, Methodology, Formal analysis. **Emelie Dahlin:** Writing – review & editing, Methodology, Formal analysis. **Zacharias Mandalenakis:** Writing – review & editing, Methodology, Formal analysis.

## Declaration of competing interest

None.

## References

[1] Mandalenakis Z., Rosengren A., Skoglund K., Lappas G., Eriksson P., Dellborg M. (2017). Survivorship in children and young adults with congenital heart disease in Sweden. JAMA Intern Med.

[2] Marelli A.J., Mackie A.S., Ionescu-Ittu R., Rahme E., Pilote L. (2007). Congenital heart disease in the general population: changing prevalence and age distribution. Circulation.

[3] Liu Y., Chen S., Zühlke L. (2019). Global birth prevalence of congenital heart defects 1970–2017: updated systematic review and meta-analysis of 260 studies. Int J Epidemiol.

[4] Gatzoulis M.A., Webb G.D., Daubeney P.E.F. (2018).

[5] Webb G., Gatzoulis M.A. (2006). Atrial septal defects in the adult: recent progress and overview. Circulation.

[6] Nyboe C., Karunanithi Z., Nielsen-Kudsk J.E., Hjortdal V.E. (2018). Long-term mortality in patients with atrial septal defect: a nationwide cohort-study. Eur Heart J.

[7] Baumgartner H., De Backer J., Babu-Narayan S.V. (2020). ESC guidelines for the management of adult congenital heart disease. Eur Heart J.

[8] Botto L.D., Lin A.E., Riehle-Colarusso T., Malik S., Correa A. (2007). National birth defects prevention study. Seeking causes: classifying and evaluating congenital heart defects in etiologic studies. Birth Defects Res A Clin Mol Teratol.

[9] Nyboe C., Olsen M.S., Nielsen-Kudsk J.E., Hjortdal V.E. (2015). Atrial fibrillation and stroke in adult patients with atrial septal defect and the long-term effect of closure. Heart.

[10] Udholm S., Nyboe C., Karunanithi Z. (2019). Lifelong burden of small unrepaired atrial septal defect: results from the Danish National Patient Registry. Int J Cardiol.

[11] Udholm S., Nyboe C., Dantoft T.M., Jørgensen T., Rask C.U., Hjortdal V.E. (2019). Small atrial septal defects are associated with psychiatric diagnoses, emotional distress, and lower educational levels. Congenit Heart Dis.

[12] Fredriksen P.M., Veldtman G., Hechter S. (2001). Aerobic capacity in adults with various congenital heart diseases. Am J Cardiol.

[13] Kempny A., Dimopoulos K., Uebing A. (2012). Reference values for exercise limitations among adults with congenital heart disease. Relation to activities of daily life—single centre experience and review of published data. Eur Heart J.

[14] Diller G.P., Dimopoulos K., Okonko D. (2005). Exercise intolerance in adult congenital heart disease: comparative severity, correlates, and prognostic implication. Circulation.

[15] Udholm S., Rex C., Eckerstrom F., Onat M., Nyboe C., Hjortdal V.E. (2019). Small unrepaired atrial septal defects display impaired exercise capacity compared with healthy peers. Congenit Heart Dis.

[16] Sandberg C., Thilen U., Wadell K., Johansson B. (2015). Adults with complex congenital heart disease have impaired skeletal muscle function and reduced confidence in performing exercise training. Eur J Prev Cardiol.

[17] Greutmann M., Le T.L., Tobler D. (2011). Generalised muscle weakness in young adults with congenital heart disease. Heart.

[18] Kröönström L.A., Johansson L., Zetterström A.-K., Dellborg M., Eriksson P., Cider A. (2014). Muscle function in adults with congenital heart disease. Int J Cardiol.

[19] Larsson L., Rinnström D., Sandberg C. (2019). Aerobic capacity in adolescence is associated with time to intervention in adult men with atrial septal defects. Int J Cardiol.

[20] White P., Myers M. (1921). The classification of cardiac diagnosis. JAMA.

[21] Kraemer W., Fleck S. (2007).

[22] Katch V.L., McArdle W.D., Katch F.I. (2011).

[23] World Health Organization (1968). Exercise tests in relation to cardiovascular function: report of a WHO meeting [held in Geneva from 25 to 30 September 1967]. World Health Organ Tech Rep Ser.

[24] Andersson A., Lundahl F., Cider Å., Dellborg M., Ashman Kröönström L. (2023). Functional muscle power in the lower extremity in adults with congenital heart disease. Int J Cardiol Congenit Heart Dis.

[25] Ashman Kröönström L., Cider Å., Zetterström A.-K. (2020). Exercise capacity, physical activity, and health-related quality of life in adults with CHD. Cardiol Young.

[26] Hébert-Losier K., Wessman C., Alricsson M., Svantesson U. (2017). Updated reliability and normative values for the standing heel-rise test in healthy adults. Physiotherapy.

[27] Cider A., Carlsson S., Arvidsson C., Andersson B., Sunnerhagen K.S. (2006). Reliability of clinical muscular endurance tests in patients with chronic heart failure. Eur J Cardiovasc Nurs.

[28] Csuka M., McCarty D.J. (1985). Simple method for measurement of lower extremity muscle strength. Am J Med.

[29] Mathiowetz V., Kashman N., Volland G., Weber K., Dowe M., Rogers S. (1985). Grip and pinch strength: normative data for adults. Arch Phys Med Rehabil.

[30] Ruuska J., Sommansson J., Svantesson U., Magnusson E., Hultenheim Klintberg I. (2005).

[31] Borg G. (1998).

[32] Strandell T. (1964). Circulatory studies on healthy old men. With special reference to the limitation of the maximal physical working capacity. Acta Med Scand Suppl.

[33] Brudin L., Jorfeldt L., Pahlm O. (2014). Comparison of two commonly used reference materials for exercise bicycle tests with a Swedish clinical database of patients with normal outcome. Clin Physiol Funct Imaging.

[34] Craig C.L., Marshall A.L., Sjostrom M. (2003). International physical activity questionnaire: 12-country reliability and validity. Med Sci Sports Exerc.

[35] World Health Organization. Body mass index (BMI). World Health Organization. Accessed December 18, 2024. https://www.who.int/data/gho/data/themes/topics/topic-details/GHO/body-mass-index.

[36] Giardini A., Donti A., Formigari R. (2004). Determinants of cardiopulmonary functional improvement after transcatheter atrial septal defect closure in asymptomatic adults. J Am Coll Cardiol.

[37] Oelberg D.A., Marcotte F., Kreisman H., Wolkove N., Langleben D., Small D. (1998). Evaluation of right ventricular systolic pressure during incremental exercise by Doppler echocardiography in adults with atrial septal defect. Chest.

[38] Stout K.K., Daniels C.J., Aboulhosn J.A. (2019). 2018 AHA/ACC guideline for the management of adults with congenital heart disease: executive summary: a report of the American College of Cardiology/American Heart Association task force on clinical practice guidelines. J Am Coll Cardiol.

[39] Pelliccia A., Sharma S., Gati S. (2021). 2020 ESC guidelines on sports cardiology and exercise in patients with cardiovascular disease [published correction appears in. Eur Heart J.

[40] Barry V.W., Baruth M., Beets M.W., Durstine J.L., Liu J., Blair S.N. (2014). Fitness vs. fatness on all-cause mortality: a meta-analysis. Prog Cardiovasc Dis.

[41] Verdicchio C., Freene N., Hollings M., Maiorana A., Briffa T., Gallagher R. (2023). A clinical guide for assessment and prescription of exercise and physical activity in cardiac rehabilitation. A CSANZ position statement. Heart Lung Circ.

