# Supplementary material for: Physical fitness in adults with isolated secundum atrial septal defects
Source: Int J Cardiol Congenit Heart Dis. 2025 Oct 24;22:100632. doi: 10.1016/j.ijcchd.2025.100632 (PMC12681933; doi:10.1016/j.ijcchd.2025.100632)
Supplement: Multimedia component 1 [file mmc1.docx]

**Supplementary appendix**

**Supplementary Table 1.** Characteristics of patients with secundum ASD according to the absence or presence of repair

| Variable | All patients with ASD | Patients who have undergone corrective surgery | | Patients who have undergone device closure | Nonoperated patients | p-value |
| --- | --- | --- | --- | --- | --- | --- |
|  | n=102 | n=38 | | n=32 | n=32 |  |
| Age, in years, median [IQR] | 43.5 [29.7, 61.0] | 40.5 [30.0, 56.5] | 47.5 [36.5, 60.7] | | 39.0 [24.2, 67.5] | 0.50^a^ |
| Sex, female | 73 (71.6) | 28 (73.7) | 22 (68.8) | | 23 (71.9) | 0.61^b^ |
| NYHA I/II/III–IV, n | 88/14/0 | 33/5/0 | 29/3/0 | | 26/6/0 | 0.55^b^ |
| Height, cm | 170.2 (9.6) | 169.7 (9.7) | 171.0 (10.4) | | 170.0 (9.0) | 0.85^c^ |
| Weight, kg, median [IQR] | 72.0 [64.7, 84.2] | 68.5 [62.7, 81.2] | 75.0 [69.2, 87.7] | | 74.0 [64.2, 88.5] | 0.25^a^ |
| BMI, kg/m^2^, median [IQR] | 25.2 [22.5, 28.6] | 24.4 [22.4, 27.5] | 26.4 [22.8, 30.3] | | 25.2 [23.1, 29.5] | 0.31^a^ |
| Systolic BP, mm Hg | 121.9 (17.8) | 119.2 (17.0) | 126.4 (17.6) | | 120.5 (18.5) | 0.21^c^ |
| Diastolic BP, mm Hg | 71.1 (9.7) | 68.8 (8.4) | 74.2 (10.2) | | 70.5 (10.0) | 0.06^c^ |
| Corrective procedure* | 70 (68.6) | 38 (100) | 32 (100) | | - |  |
| Device closure | 39 (38.2)** | 7 (18.4) | 32 (100) | | - |  |
| Sternotomy | 35 (34.3) | 35 (92.1) | - | | - |  |
| Thoracotomy | 4 (3.9) | 4 (10.5) | - | | - |  |
| Total number of sternotomies/thoracotomies | 1.03 (1.16) | 1.03 (1.16) | - | | - |  |
| Age at first corrective procedure, years |  |  |  | |  |  |
| ≤5 | 4 (3.9) | 4 (10.5) | 0 (0.0) | | - |  |
| 6–17 | 12 (11.8) | 12 (31.6) | 0 (0.0) | | - |  |
| ≥18 | 54 (52.9) | 22 (57.9) | 32 (100) | | - |  |
| Atrial fibrillation, yes | 18 (17.6) | 6 (15.8)*** | 6 (18.8) | | 6 (18.8) | 0.93^b^ |
| Atrial flutter, yes | 6 (5.9) | 4 (10.5) | 2 (6.3) | | 0 (0.0) | 0.17^b^ |
| Cardiovascular medication, yes | 53 (52.0) | 16 (42.1) | 23 (71.9) | | 14 (43.8) | 0.02^b^ |
| Acetylsalicylic acid | 26 (25.5) | 4 (10.5) | 16 (50.0) | | 6 (18.8) |  |
| Anticoagulant | 15 (14.7) | 6 (15.8) | 4 (12.5) | | 5 (15.6) |  |
| Antiarrhythmia | 4 (3.9) | 1 (2.6) | 1 (3.1) | | 2 (6.3) |  |
| Blockade of RAAS | 14 (13.7) | 5 (13.2) | 3 (9.4) | | 6 (18.8) |  |
| Beta blocker | 26 (25.5) | 8 (21.1) | 9 (28.1) | | 9 (28.1) |  |
| Calcium antagonist | 6 (5.9) | 2 (5.3) | 1 (3.1) | | 3 (9.4) |  |
| Diuretic | 9 (8.8) | 2 (5.3) | 1 (3.1) | | 6 (18.8) |  |
| Statin | 6 (5.9) | 0 (0.0) | 3 (9.4) | | 3 (9.4) |  |
| Other | 2 (2.0) | 0 (0.0) | 0 (0.0) | | 2 (6.3) |  |
| IPAQ****  Low/Moderate/High | n=74  20/36/18 | n=27  8/15/4 | n=21  3/10/8 | | n=26  9/11/6 | 0.28^b^ |
| MET (min/week), median [IQR] | 1404 [693.0, 2958.0] | 1044 [594.0, 2160.0] | 2224 [693.0, 5532.0] | | 1431 [805.5, 2912.2] | 0.19^a^ |

Data are given as n (%) or mean (standard deviation [SD]) unless otherwise noted. ASD: atrial septal defects, NYHA: New York Heart Association, BMI: body mass index, BP: blood pressure, RAAS: Renin-angiotensin-aldosterone system, IPAQ: International Physical Activity Questionnaire, MET: Metabolic Equivalent of Task, IQR: interquartile range.

*Some patients underwent multiple procedures.

**n=4 – had undergone device closure ≤1 year prior to corrective surgery and thus considered corrective surgery only.

***n=1 – had a diagnosis of both atrial fibrillation and atrial flutter.

****Not all patients filled in the IPAQ, resulting in missing variables.

^a^Kruskal–Wallis Test

^b^Chi-square test

^c^ANOVA
